# Supplementary material for: Antagonism of Rhizosphere Streptomyces yangpuensis CM253 against the Pathogenic Fungi Causing Corm Rot in Saffron (Crocus sativus L.)
Source: Pathogens. 2022 Oct 16;11(10):1195. doi: 10.3390/pathogens11101195 (PMC9607649; doi:10.3390/pathogens11101195)
Supplement: Supplementary file 1 [file pathogens-11-01195-s001.zip › pathogens-1914195-supplementary.pdf]

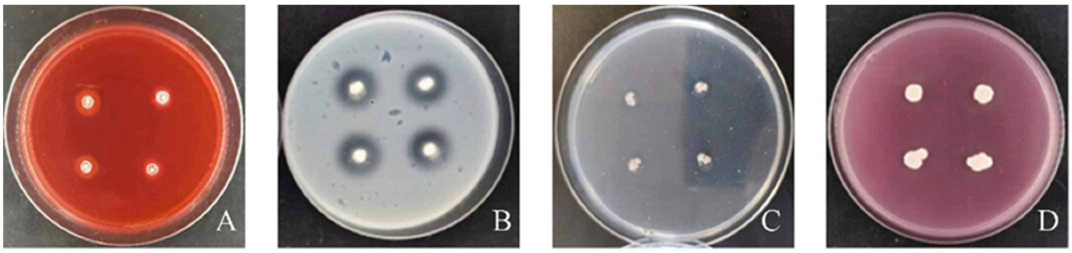

Figure S1. Hydrolytic enzymes' activities. A: cellulase +, B: protease +, C: chitinase –, and D: glucanase +.

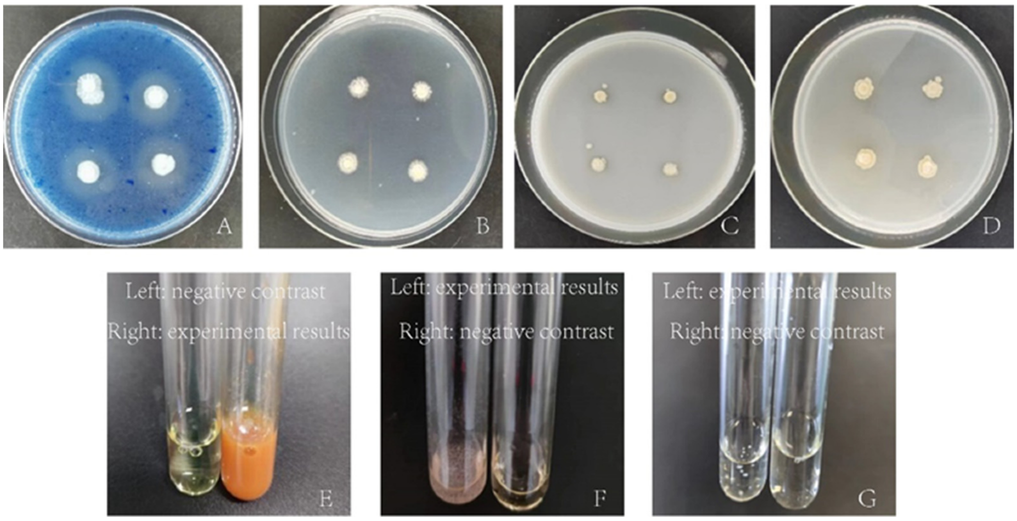

Figure S2. Plant-growth-promoting attributes. A: siderophore production +; B: potassium dissolution–; C: inorganic phosphate solubilization –; D: organic phosphate solubilization –; E: NH_3 production +; F: ACC deaminase enzyme +; and G: IAA production +.
